# Discrepancy between radiography and magnetic resonance imaging in Japanese Investigation Committee classification type C osteonecrosis of the femoral head

**DOI:** 10.1007/s00264-024-06396-x

**Published:** 2024-12-26

**Authors:** Keiji Otaka, Yusuke Osawa, Yasuhiko Takegami, Hiroto Funahashi, Shiro Imagama

**Affiliations:** Nagoya University, Nagoya, Japan

**Keywords:** Radiography, Magnetic resonance imaging, Femoral head osteonecrosis

## Abstract

**Purpose:**

The Japanese Investigation Committee (JIC) classification for osteonecrosis of the femoral head (ONFH) is based on the necrotic area relative to the weight-bearing surface on anteroposterior (AP) radiographs or central coronal MRI. Discrepancies exist between these methods, potentially related to the AP necrosis area. This study evaluated these discrepancies and the extent of AP necrotic lesions.

**Methods:**

We retrospectively reviewed 139 patients (188 hips) with nontraumatic ONFH, JIC type C1 or C2 on radiography, and collapse < 3 mm. Cases with and without discrepancies between radiography and MRI were designated as discrepancy and consistent groups, respectively. We assessed the proportion of patients in the discrepancy group and survival rates in both groups, with femoral head collapse > 3 mm as the endpoint. The cutoff value for AP necrotic regions on lateral radiographs identifying discrepancies was calculated using ROC curve analysis.

**Results:**

The discrepancy group comprised 28 hips (14.9%) vs. 160 hips in the consistent group. Five-year survival rates were 73.3% vs. 31.9% (*P* < 0.01), and AP necrotic region extent was 61.2 vs. 73.8 mm (*P* < 0.001) in discrepancy vs. consistent groups. The cutoff value for necrotic region extent revealing discrepancies was 66.9% (AUC 0.833, sensitivity 83.8%, specificity 82.4%).

**Conclusion:**

Patients with AP necrotic regions < 66.9% were more likely to show discrepancies between radiography and MRI in type classification. This study can help improve accuracy in assessing ONFH severity and prognosis.

## Introduction

Osteonecrosis of the femoral head (ONFH) is a progressive hip joint disorder that can lead to femoral head collapse and joint destruction [1, 2]. Collapse causes pain and often requires surgical intervention. Larger necrotic areas are associated with a higher likelihood of collapse [3–5]. Therefore, accurate assessment of the necrotic region of the femoral head is crucial for planning treatment. Various classifications have been used to evaluate the extent of necrosis in order to predict the prognosis of ONFH [6–8]. The Japanese Investigation Committee (JIC) classification is relatively easy to use and is characterized by minimal interobserver variability [9, 10]. According to the JIC classification, type C presents with necrosis on more than two-thirds of the weight-bearing surface, and is generally known to have a high collapse rate and poor prognosis [4, 5, 11].

The JIC-type classification is evaluated based on the necrotic area relative to the weight-bearing surface in the anteroposterior (AP) radiographic view or the central coronal section of the femoral head on magnetic resonance imaging (MRI) T1-weighted images [6]. In clinical practice, there are a few cases in which discrepancies in the JIC-type classification are observed between radiography and MRI. However, the frequency of discrepancies between the two methods is unknown. Additionally, the presence or absence of discrepancies may affect the prognosis of femoral head collapse. The localization of necrosis is considered a factor that causes these discrepancies in image evaluation. In many cases of ONFH, the necrotic area is located in the anterosuperior region of the femoral head [12]. We hypothesized that the extent of AP necrotic lesions of the femoral head is related to discrepancies in the JIC-type classification between the two examination methods. The purpose of this study was to evaluate the discrepancies in type classification between the two modalities and the extent of AP necrotic lesions of the femoral head.

In this study, we asked the following questions regarding JIC type C, which has a high risk of collapse: (1) What is the proportion of discrepancies between radiographic and MRI evaluations? (2) Do these discrepancies in the JIC-type classification due to different imaging evaluation methods affect the collapse rate of the femoral head? (3) Is there a cutoff value for the extent of AP necrotic lesions that helps identify discrepancies in the JIC type classification?

## Materials and methods

The study was approved by the ethics committee of our institution and performed in line with the principles of the Declaration of Helsinki. We retrospectively reviewed 234 patients (370 hips) with nontraumatic ONFH who visited our hospital between January 2013 and December 2020. The following cases were excluded: (1) cases classified as JIC type A or B based on radiographic images, (2) early-stage cases without bone sclerosis that could not be diagnosed as ONFH by radiography alone, and (3) cases with a collapse >3 mm at initial examination. After excluding 182 hips, 139 patients (188 hips) who had JIC type C based on radiography, those with a collapse of <3 mm were included in this study. There were 82 men and 57 women, with a mean age of 44.1 years and a mean follow-up period of 48.2 months (Table 1).

**Table 1:** Demographics of the patients

Number of patients	139
Age (year old)	44.1 (16 to 81)
Sex (men/women)	82 / 57
BMI (kg/m^2^)	22.0 (14.9 to 28.6)
Mean follow-up (months)	48.2 (1 to 126)
Associated risk factors (steroid/alcohol/idiopathic)	90 / 38 / 11

BMI, body mass index

We investigated the incidence of discrepancies in image evaluations between radiography and MRI. The 188 hips from 139 patients were classified according to the JIC classification for each imaging modality. Patients who did not have discrepancy in type classification between radiography and MRI were designated as the consistent group, whereas those with discrepancy were designated as the discrepancy group. We also evaluated the following characteristics: age, sex, body mass index (BMI), associated factors, joint survival rate with radiographic failure as the endpoint, and extent of the necrotic region in lateral radiographs of the hip joint for both groups.

### Classification methods

**Fig. 1:**
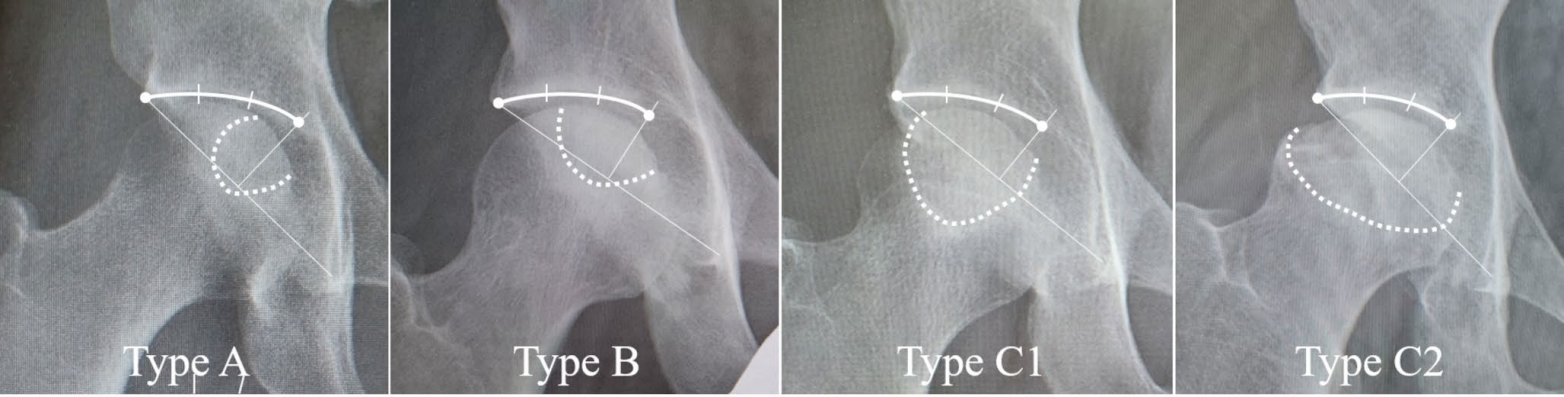
The Japanese Investigation Committee classification. Type A regions occupy the medial third or less of the weight-bearing area. Type B regions occupy the medial two-thirds or less of the weight-bearing area. Type C1 and C2 lesions both occupy more than the medial two-thirds of the weight-bearing area, but, whereas type C2 lesions extend laterally to the acetabular edge, type C1 lesions do not

The JIC classification was performed as previously described [6]. Necrotic lesions were classified based on their location relative to the weight-bearing surface in the anteroposterior (AP) radiographic view or the central coronal section of the femoral head on MRI T1-weighted images. Type A was defined as a necrotic area less than the medial one-third of the weight-bearing area. Type B was defined as a necrotic area less than medial two-thirds of the weight-bearing area. Type C1 was defined as a necrotic area exceeding the medial two-thirds of the weight-bearing area but medial to the lateral edge of the acetabulum. Type C2 was defined as a necrotic area exceeding the medial two-thirds of the weight-bearing area and extending laterally to the acetabular edge. The weight-bearing area was defined as the area lateral to the mid-vertical line of the line through the acetabular edge and the teardrop bottom. (Fig. 1).

### Image evaluations

**Fig. 2:**
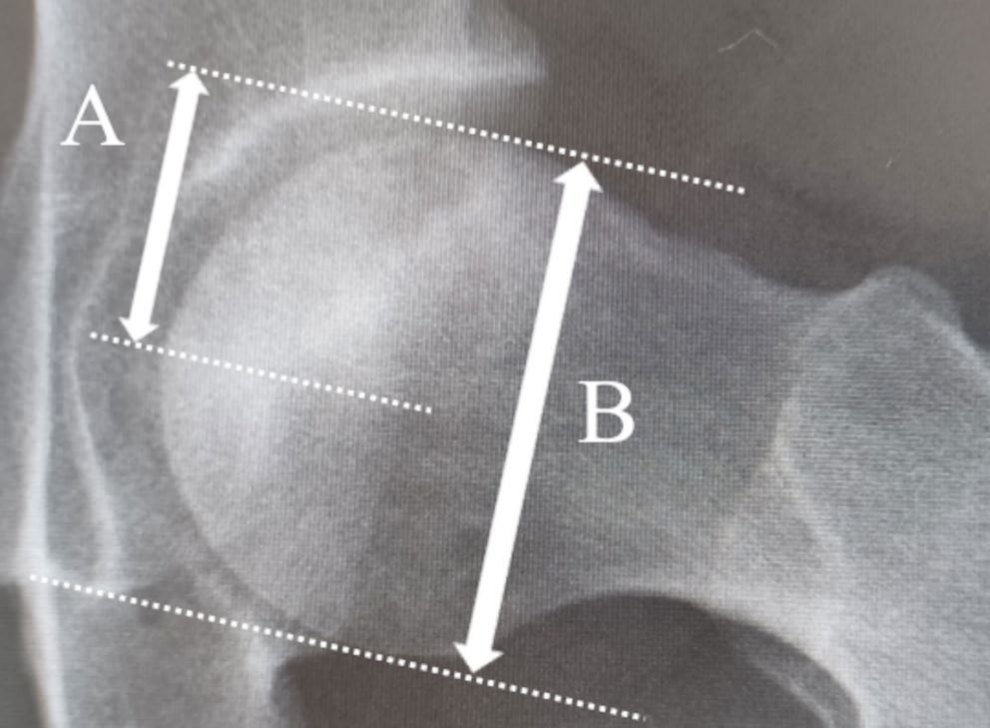
The extent of the anteroposterior (AP) necrotic lesions was measured as the ratio of the longest AP length of the necrotic lesion (**A**) to the largest AP diameter of the femoral head (**B**) on lateral radiograph

Femoral head collapse was evaluated according to previously reported methods, with radiographic failure defined as a collapse of >3 mm on AP and lateral hip joint images [13, 14]. This collapse was measured as the distance between the maximum incursion into the femoral head and the estimated circle outline in both anteroposterior and lateral radiographs. Radiographs were taken every six months from the initial examination to assess femoral head collapse. Radiographic evaluation of the degree of collapse was performed using a HOPE/EGMAINGX computer system (Fujitsu Ltd., Tokyo, Japan) at a magnification of 400%. The extent of AP necrotic regions in the femoral head was evaluated based on methods reported by Nam et al. [15]. Briefly, these were measured as the ratio of the longest AP length of the necrotic regions to the diameter of the femoral head on the lateral hip joint images (Fig. 2). To assess interobserver reliability, two surgeons randomly selected 50 cases and evaluated the radiographic collapse and AP necrotic regions twice. The intra-observer reliability values for the radiographic collapse and AP necrotic regions were 0.89 and 0.91, respectively.

### Data analyses

Shapiro–Wilk tests were performed to determine the distributions of data. For normally distributed variables, Student’s t-test was used to compare the groups. When variables were not normally distributed, Mann–Whitney U test was used to compare the groups. The chi-square test was used for categorical variables. Survival rates with a collapse of > 3 mm as the endpoint and comparisons between the two groups were evaluated using the Kaplan-Meier method and log-rank test, respectively. For survival analysis, 26 hips that received joint-preserving treatment and 54 hips from 43 patients who had a follow-up period of <two years were excluded, resulting in an analysis of 108 hips. The cutoff value for the AP-necrotic region was calculated using a receiver operating characteristic (ROC) curve. All statistical analyses were performed using the EZR software program (Saitama Medical Center, Jichi Medical University), and *p* < 0.05 were considered statistically significant [16].

## Results

The JIC classification, according to radiographic evaluation, was as follows: type C1 and type C2 in 81 and 107 hips, respectively. The JIC classification according to MRI evaluation was as follows: type B, type C1 and type C2 in 11, 87 and 90 hips, respectively. Discrepancies in the JIC-type classification between radiography and MRI were observed in 28 patients (14.9%). Consequently, discrepancy group comprised 28 hips, and consistent group comprised 160 hips.

**Fig. 3:**
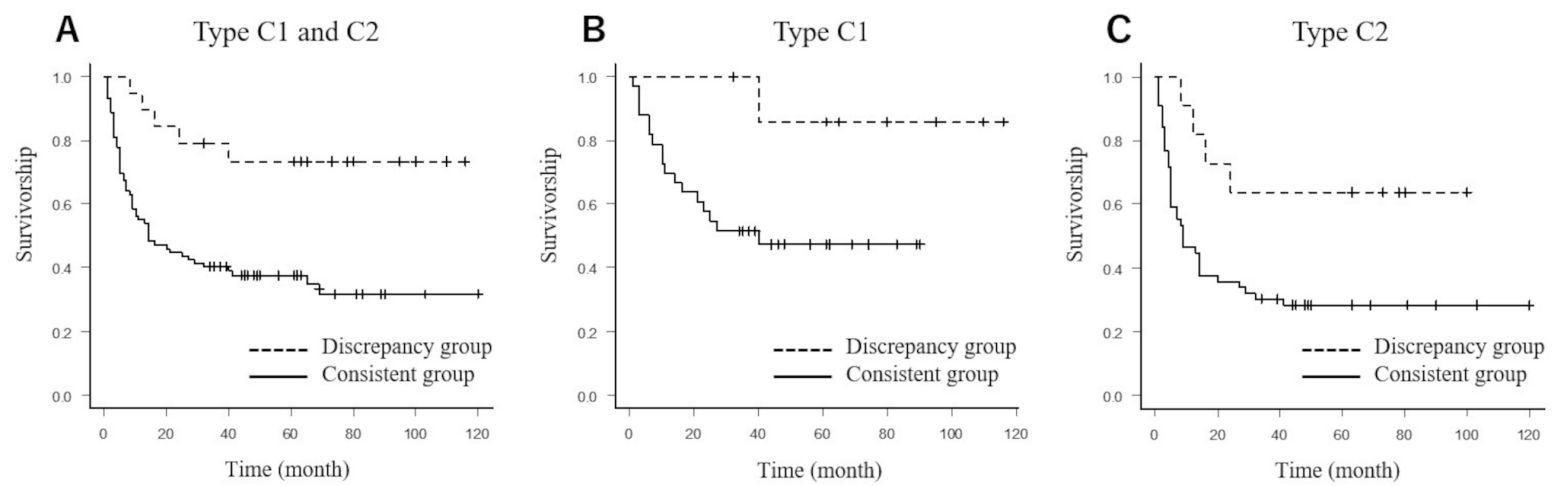
The comparative survival rates with collapse > 3 mm as the endpoint between the consistent group and the discrepancy group in type C1 and C2 (**A**), type C1 (**B**) and type C2 (**B**)

No significant differences were observed between the two groups in terms of age, sex, BMI, associated factors, or JIC stage classification (Table 2). The overall five year survival rate (108 hips) with collapse > 3 mm as the endpoint was 73.3% in discrepancy group and 31.9% in consistent group, showing a significant difference (*P* = 0.0024). When limited to type C1 (41 hips), the survival rate with collapse > 3 mm as the endpoint was 85.7% in discrepancy group and 47.2% in consistent group, demonstrating a significant difference (*P* = 0.048). Similarly, for Type C2 (67 hips), the survival rate with collapse > 3 mm as the endpoint was 63.6% in discrepancy group compared with 28.3% in consistent group, also showing a significant difference (*P* = 0.026) (Fig. 3).

**Table 2:** Demographics and Extent of the Necrotic Regions of the Discrepancy group and the Consistent group

	Discrepancy group (*n* = 28)	Consistent group (*n* = 160)	*P* value
Age (year old)	40.6 (23 to 55)	44.3 (16 to 81)	0.211
Sex (men/women)	18/10	92/68	0.54
BMI (kg/m^2^)	21.6 (18.3 to 27.3)	22.1 (14.9 to 28.6)	0.503
Associated risk factors (steroid/alcohol/idiopathic)	16/9/3	111/38/11	0.432
JIC stage classification (2/3A)	11/17	47/113	0.375
Extent of necrotic region (%)	61.5 ± 9.8	72.8 ± 9.1	< 0.001

BMI, body mass index; JIC, Japanese Investigation Committee

**Fig. 4:**
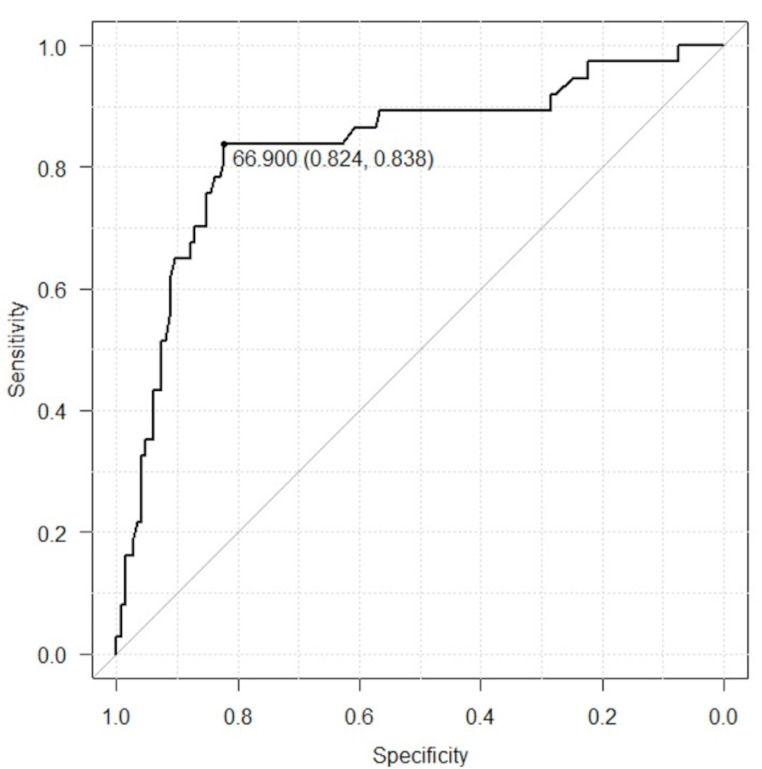
The receiver operating characteristic curve of the extent of anteroposterior necrotic region that would help identify discrepancies in type classification between radiography and MRI

The extent of the AP necrotic regions on lateral radiographs was significantly different between the discrepancy and consistent groups (61.5 vs. 72.8 mm) (*P* < 0.001) (Table 2). The cutoff value for the AP necrotic region that would help identify discrepancies in type classification between radiography and MRI was calculated using the ROC curve. The area under the curve was 0.833, and the cutoff value was 66.9% (sensitivity, 83.8%; specificity, 82.4%) (Fig. 4).

## Discussion

**Fig. 5:**
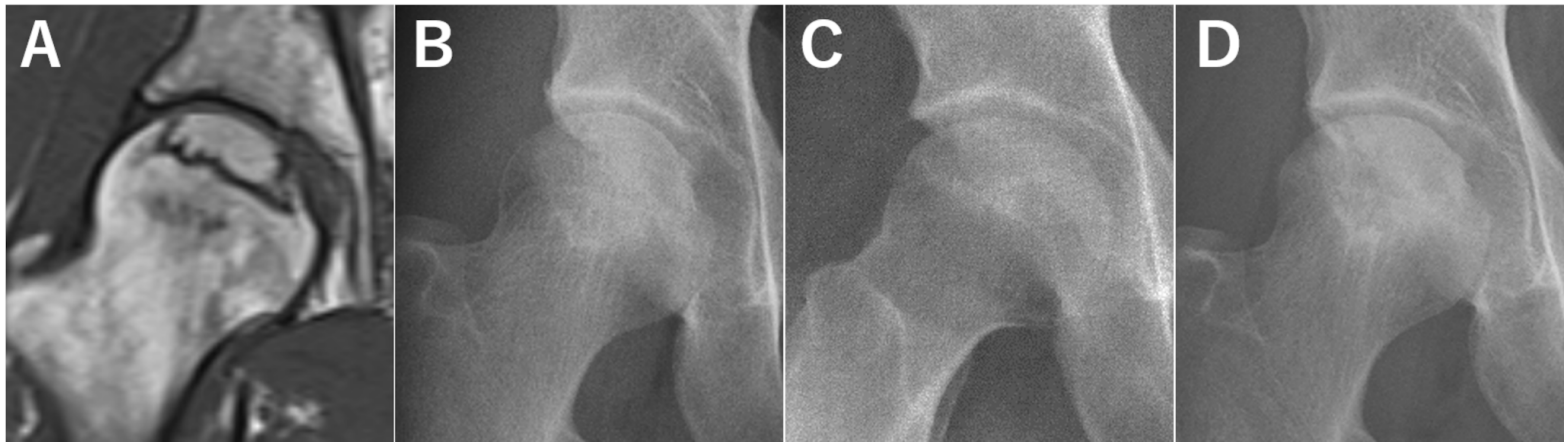
Osteonecrosis of femoral head of a 28-year-old man with discrepancy in the Japanese Investigation Committee type classification between radiography and magnetic resonance imaging (MRI). (**A**) The central coronal section of the femoral head on MRI T1-weighted image, it is classified as type C1. (**B**) The anteroposterior (AP) radiographic view of the femoral head, it is classified as type C2. (**C**) The lateral radiographic view. The extent of necrotic area is located anteriorly, and the extent of the AP necrotic region is 60%. (**D**) AP radiograph obtained 5 years after the first visit shows no collapse progression of the right femoral head

In this study, we observed discrepancies in the JIC classification between radiographic and MRI evaluations in 14.9% of ONFH cases. Cases showing discrepancies in type classification demonstrated a more favourable prognosis for femoral head collapse. It was found that the extent of the AP necrotic regions was associated with these discrepancies, and the cutoff value for the AP necrotic regions that aided the identification of discrepancies was 66.9% (Fig. 5).

The JIC classification system proposed in Japan, is now used worldwide to classify ONFH. Takashima et al. reported that compared to the Kerboul and Steinberg classifications, the JIC classification has smaller interobserver variability and is a highly versatile and effective classification system [9]. Although the evaluation method involves the use of either AP radiographs of the hip joint or MRI coronal sections at the central level of the femoral head, there is no defined priority between these imaging modalities. Depending on the localization of necrosis, discrepancies may arise in the decision of type classification between radiography and MRI, potentially leading to misjudgment of the collapse risk. The results of this study showed discrepancies in the JIC-type classification between radiography and MRI in 28 (14.9%) of 188 joints. Although type classification based on plain radiography is a highly versatile approach, surgeons should be aware of the possibility of discrepancies with the MRI-based classifications.

Most large-scale studies on survival by type in the JIC classification have been based on MRI. Asada et al. reported collapse rates at 36 months for each JIC type as follows: type A, 0%; type B, 10.8%; type C1, 48.5%; and type C2, 76.7% [17]. Similarly, Kuroda et al. reported five year collapse rates for types A (0%), B (7.9%), C1 (36.6%), and C2 (84.8%) [18]. Our results showed that patients who showed discrepancies between radiography and MRI exhibited significantly better survival rates, with a collapse of > 3 mm as the endpoint, than those without discrepancies. Therefore, for cases showing discrepancies, MRI-based classification may be more appropriate than radiography-based classification for evaluating survival rate.

Necrotic lesions of ONFH are generally located anteriorly [12]. Furthermore, the extent of AP necrosis in ONFH has been shown to significantly affect prognosis. Osawa et al. reported that the extent of AP necrotic regions in the femoral head was associated with the cessation of femoral head collapse [19]. They found that the prognosis was significantly better with collapse progression as the endpoint when the AP necrotic region on MRI was < 62.1%. Ikemura et al. investigated the posterior boundary of necrotic lesions in ONFH and reported that cases with progressive collapse had significantly larger necrotic angles in the axial and oblique axial slices on MRI than cases with non-progressive collapse [20]. In our study, we found that the cutoff value for the AP necrotic region on lateral hip radiographs for predicting discrepancies in type classification between radiographs and MRI was 66.9%. Cases with AP necrotic regions < 66.9% on lateral radiographs may be overestimated in the type classification and should be further evaluated using MRI.

This study has several limitations. First, this was a retrospective study. A prospective investigation is necessary to validate our findings regarding the progression of collapse. Second, our study was limited to type C radiographs and may not be applicable to other types. Furthermore, the survival rates obtained in this study did not consider risk factors for collapse, such as necrotic size or volume. Additionally, the cutoff value obtained in this study was used to predict discrepancies in type classification between radiography and MRI, but it did not directly predict prognosis. Nevertheless, this study had several strengths. MRI cannot be performed in all cases in clinical practice. The threshold of the AP necrotic region in the lateral hip radiograph identified in this study can be used to identify cases that should be evaluated using MRI. Therefore, the results of this study may help determine treatment strategies for ONFH.

In conclusion, there were discrepancies in the JIC-type classification between radiography and MRI in 14.9% of ONFH type C cases. Patients who had with discrepancies demonstrated significantly better survival rates with femoral head collapse > 3 mm as the endpoint than those without discrepancies. The cutoff value for the AP necrotic region on lateral hip radiographs that aided the identification of discrepancies in type classification between radiography and MRI was 66.9%. These results may be useful in determining a treatment plan for ONFH.

## Data Availability

No datasets were generated or analysed during the current study.
